# Insulin-Like Growth Factors in Development, Cancers and Aging

**DOI:** 10.3390/cells9102309

**Published:** 2020-10-17

**Authors:** Haim Werner

**Affiliations:** Department of Human Molecular Genetics and Biochemistry, and the Shalom and Varda Yoran Institute for Human Genome Research, Sackler School of Medicine, Tel Aviv University, Tel Aviv 69978, Israel; hwerner@tauex.tau.ac.il

Since their discovery in the late 1950s, insulin-like growth factors (IGFs) have attracted significant interest in multiple areas of biology and medicine, including endocrinology, pediatrics, growth, metabolism, nutrition, aging, and oncology. IGF1, which was initially identified as the mediator of growth hormone (GH) action, is regarded as a key player in numerous cellular and organismal processes. The signaling pathways elicited by IGF1 have been extensively characterized in biochemical and molecular terms over the past 40 years. However, fundamental questions regarding basic differences between the mechanisms of action of IGF1 and the closely related insulin molecule are yet to be resolved. This Special Issue of *Cells* provides a collection of modern articles dealing with the role of IGF1 in cancer biology, aging, and development. The articles explore basic and clinical aspects of the IGF1 system, including post genomic analyses as well as novel approaches to target the IGF1R in oncology.

The role of IGF binding proteins (IGFBPs) in the regulation of IGF1-stimulated growth has been the focus of intensive research for many years. Forced expression of IGFBPs in transgenic mice, under most circumstances, leads to inhibitory effects on somatic growth. To evaluate the impact of IGFBPs on normal growth, Walz et al. [1] measured IGF1 and IGFBP-2, -3, and -4 levels in the serum of growth-selected mouse models (obese and lean) and expressed these values as a function of longitudinal growth. The authors provide evidence that part of the elevated growth activity during prepubertal growth could be related to the elevated bioactivity of IGF1. Specifically, elevated ratios of IGF1/IGFBPs were established by a delayed increase in IGFBPs compared to a strong increase in IGF1 levels between two and four weeks of age.

As mentioned above, the IGF1 axis plays a key role in aging and longevity. However, the biochemical and molecular mechanisms responsible for the linkage between IGF1 and aging processes are poorly defined. Zhang et al. [2] evaluated age- and sex-adjusted hazards for all-cause mortality and incident age-related diseases in a prospective cohort of older adults (mean age = 76.1 ± 6.8 year) as predicted by baseline total serum IGF1, IGFBP-1, IGFBP-3, and IGF1/IGFBP-3 molar ratio. The authors report that higher IGF1 levels and bioavailability predicted mortality and morbidity risk, supporting the hypothesis that diminished GH-IGF1 signaling may contribute to human longevity and health-span.

Yoshida and Delafontaine [3] provide a comprehensive review of the role of IGF1 and its downstream signaling paths in skeletal muscle atrophy associated with chronic diseases and aging. The authors describe the involvement of autophagy in IGF1-stimulated muscle atrophy and protein degradation. In addition, they emphasize the fact that given the multiple (sometimes opposed) interactions of IGF1 in skeletal muscle, it is often difficult to clearly define the specific role of IGF1 in this tissue. The authors conclude that further studies are required to develop effective strategies to apply IGF1 in order to treat muscle atrophy in humans.

Glucose regulated protein 94 (GRP94) is a ubiquitously expressed chaperone in the endoplasmic reticulum that is required for the proper folding and secretion of IGF1. Argon et al. [4] review the implications of IGF1–GRP94 interaction in the context of idiopathic short stature and suggest that the chaperone machinery can be modulated with small molecules. The net result of this molecular intervention might constitute a novel way to manipulate both IGF1 deficiency as well as conditions of excessive growth factor production. Similarly, IGF1–GRP94 interaction might be relevant in cancer. Thus, differences in the association of IGF1/IGF2 with GRP94 can be exploited for selective tissue targeting of compounds.

Ahmad et al. [5] describe the role of IGF1 in the maintenance of skeletal muscle mass. Specifically, their review focuses on the mechanisms involved in the proliferation of muscle satellite cells as well as the key role of IGF1 in myoblast differentiation during normal growth or regeneration after skeletal muscle injury. The authors state that the development of protocols for the use of IGF1 in muscle-wasting conditions remains an important research challenge.

The mitochondria are key organelles that regulate vital processes in eukaryotic cells. A decline in mitochondrial function is regarded as an important hallmark of aging. Poudel et al. [6] review the evidence that GH and IGF1 regulate mitochondrial mass and function and contribute to specific processes of cellular aging. The authors highlight the involvement of these hormones in mitochondrial biogenesis, ATP production, oxidative stress, and senescence, with a special focus on mitochondrial pathologies during aging.

IGFBP-3 is the best characterized IGF binding protein and its disruption has been linked to a number of pathologies. IGFBP-3 exhibits a number of IGF1-independent activities, ranging from tumor suppressing to tumor promoting effects. Cai et al. [7] describe the identification of TMEM219, an unknown transmembrane protein, as a potential IGFBP-3 interacting protein. Furthermore, they delineate the underlying mechanisms and biological implications of IGFBP-3–TMEM219 interplay. Finally, the authors portray the therapeutic potential of TMEM219 agonists for cancer therapy.

As alluded to above, the IGF1R constitutes a promising target in oncology. Chen et al. [8] review the current state of IGF-targeting approaches and outline the stepwise bioengineering and validation of *IGF-Trap*, a novel anticancer modality that could bypass the limitations of current techniques, including interference with insulin receptor signaling. In vivo, IGF-Trap displays favorable kinetic properties and could reduce metastatic outgrowth of colon and lung cancers in the liver. In addition, Chen and colleagues developed a sensitive IGF kinase receptor-activation (KIRA) assay that serves as a surrogate biomarker for drug efficacy.

The inherent complexity of the IGF1 system is elegantly discussed by Janssen [9]. While the classical view postulated that phosphorylation of tyrosine residues plays a major role in IGF1R activation, there is increasing evidence showing that this dogma was too simplistic and grossly underestimated the downstream complexity of the IGF1R pathways. Janssen discusses the novel concept that IGF1R can be also considered as a functional tyrosine kinase/G-protein coupled receptor (GPCR) hybrid. According to this view, this hybrid is able to integrate kinase signaling with some IGF1R-mediated GPCR features. In summary, the IGF1R is far more complex than previously thought and a big challenge for the future will be to integrate and translate this new knowledge into clinical practice.

Finally, recent developments in the area of IGF-II research are discussed by Blyth et al. [10]. IGF-II is the least investigated ligand of the IGF system and it is unique in that it acts through both the IGF1R and the insulin receptor isoform A (IR-A). The solved structure of IGF-II bound to IGF1R using cryo-electron microscopy is clearly depicted. In addition, comparisons are made with the structures of insulin and IGF1 bound to their cognate receptors. Lastly, the authors discuss future investigations required to develop antagonists of IGF action for cancer treatment.
